# Diagnosis and Monitoring of Tunnel Lining Defects by Using Comprehensive Geophysical Prospecting and Fiber Bragg Grating Strain Sensor

**DOI:** 10.3390/s24061749

**Published:** 2024-03-08

**Authors:** Chuan Li, Jiaqi Li, Chuan Luo, Qiang Xu, Xiaorong Wan, Lubing Yang

**Affiliations:** 1 Faculty of Information Engineering and Automation, Kunming University of Science and Technology, Kunming 650500, China; 2 Yunnan Key Laboratory of Computer Technology Applications, Kunming 650500, China; 3 Yunnan Aerospace Engineering Geophysical Detecting Co., Ltd., Kunming 650200, China

**Keywords:** comprehensive non-destructive exploration, tunnel lining, carbon-fiber-strengthened concrete, stress–strain, fiber Bragg grating strain sensors

## Abstract

Tunnel excavation induces the stress redistribution of surrounding rock. In this excavation process, the elastic strain in the rock is quickly released. When the maximum stress on the tunnel lining exceeds the concrete’s load-bearing capacity, it causes cracking of the lining. Comprehensive geophysical exploration methods, including seismic computerized tomography, the high-density electrical method, and the ultrasonic single-plane test, indicated the presence of incomplete distribution of broken rock along the tunnel axis. Based on the geophysical exploration results, a carbon-fiber-strengthened tunnel simulation model was established to analyze the mechanical characteristics of the structure and provide a theoretical basis for sensor deployment. Fiber Bragg grating (FBG) strain sensors were used to measure the stress and strain changes in the second lining concrete after carbon reinforcement. Meanwhile, one temperature sensor was installed in each section to enable temperature compensation. The monitoring results demonstrated that the stress–strain of the second lining fluctuated within a small range, and the lining did not show any crack expansion behavior, which indicated that carbon-fiber-reinforced polymer (CFRP) played an effective role in controlling the structural deformation. Therefore, the combined detection of physical exploration and FBG sensors for the structure provided an effective monitoring method for evaluating tunnel stability.

## 1. Introduction

Tunnel excavation destroys the initial stress state in the surrounding rock and redistributes the stress. Due to the re-consolidation between soil layers and the change in the water flow path within the rock, the rock stress needs to be adjusted for a long time to reach a new equilibrium state [1,2,3,4]. When the redistributed stress exceeds the elastic range of the surrounding rock, it can lead to cracking of the lining. Additionally, the broken rock of the mountain may also lead to separation and cracking between the rock layers. In this case, the rock layers lose their integrity and continuity, posing a threat to the stability of the tunnel.

The direct cause of cracks in the tunnel surrounding rock and lining is the stress redistribution and geological fragmentation of rocks. Thus, in order to analyze this behavior, the first step is to clarify the tunnel engineering geological environment. Many scholars have combined multiple geophysical methods to analyze tunnel geology. To date, there have been many research results for the physical exploration of tunnels. Nie et al. [5] employed a combination of surface electrical resistivity tomography, seismic ahead-of-time exploration, and borehole resistivity imaging to assess the spatial distribution and water content. The results confirmed that the water-bearing sand layer was the main cause of tunnel disease. Huang et al. [6] found a strong radar response signal and low apparent resistivity distribution in the tunnel subsurface water-bearing structure by tunnel seismic prediction (TSP), ground penetrating radar (GPR), and the high-density electrical method. Shen et al. [7] utilized GPR and the high-density electrical method to comprehensively detect karst structures and tunnel lining diseases. The results indicated that karst formations and groundwater were the primary causes of tunnel lining cracking and the arching rise. Monitoring points are commonly located through physical exploration during the stability analysis of structures [8]. The stability of tunnel engineering during the construction period can be evaluated based on the changes in the strain of the second lining. In terms of structural monitoring, there are various types of sensor applications, including resistive strain gauges, micro-electro-mechanical system (MEMS) sensors, fiber optic sensors, piezoelectric accelerometers, etc. Fiber optic sensors have been used in large linear projects due to their low additional losses, small size, and immunity to electromagnetic interference [9,10]. Thus, fiber optic sensors are better suited than other sensors for high-precision, long-range, and multi-parameter monitoring of structural strain. Due to the different monitoring ranges and point continuity, fiber optic sensor measurements are typically divided into point-based and distributed measurement. Distributed sensing techniques such as optical frequency domain reflectometry (OFDR) and phase-sensitive optical time domain reflectometry (ϕ-OTDR) enable global strain measurements to be achieved on a single optical fiber [11]. Xu et al. [12] obtained the spatiotemporal evolution characteristics of fractures in the soil strain field through the OFDR distributed strain measurement method and established the correlation between strain and fractures. Liu et al. [13] deployed a distributed OFDR fiber sensing network to analyze the cracking behavior of concrete. The results revealed the relationship between the bond stress–slip behavior of concrete cracks. The single point measurement of optical fiber is typical of fiber Bragg grating (FBG) strain sensing, and its installation position and method are more flexible. Each FBG sensor can correspond to a specific position, and multiple sensors can be connected in series to achieve multi-point measurements. In some structures that require localized measurements, FBG sensing may be simpler and exhibit a faster response to signals. Lai et al. [14] evaluated the safety of tunnel structures by using FBG sensors to monitor the real-time strain and internal force of concrete lining in loess tunnels. The monitoring results demonstrated a clear linear characteristic of concrete strain during the early stage, gradually converging towards a stable value over time. Ding et al. [15] utilized FBG sensing technology to analyze the stress distribution of segments during shield tunnel construction. The tunnel design was optimized based on monitoring data, aiming to achieve a safer structure. Mu et al. [16] analyzed the effects of different soil parameters on lining deformation and settlement through finite element modeling, based on which distributed fiber optic sensors were installed to monitor tunnel lining deformation. Based on the monitoring data, the longitudinal strain, longitudinal curvature, longitudinal settlement, and circumferential convergence of the tunnel were analyzed. Some scholars combined physical exploration methods with FBG strain sensors to monitor the second lining of the broken section of the tunnel. Li et al. [17] identified Shan–Xinpo tunnel defects using GPR and installed differential FBG sensors to measure structural strain. By predicting the deformation trend, concrete was sprayed again to support the surrounding rock during the tunnel backfilling process, ensuring the stability and safety of the tunnel. Zhao et al. [18] utilized seismic computerized tomography (CT) to identify the geological fracture zone around the No. 3 tunnel in Bai–Nijing and employed FBG and Brillouin monitoring technologies for tunnel detection. Based on the monitoring results, an analysis was conducted to understand the reasons behind the abnormal strain of the structure during the dry and wet seasons.

Currently, the research on the integration of physical exploration methods with FBG monitoring methods is not very comprehensive. During continuous arch tunnel excavation, complex geological problems could arise. However, relying solely on a single physical exploration method does not provide sufficient and diverse information. Thus, in this paper, comprehensive geophysical prospecting methods were used in the geological survey of the tunnel. Based on the prospecting result, the carbon-fiber-reinforced polymer (CFRP) fabric was pasted on the surface of the lining concrete to strengthen it. Meanwhile, the stress environment after tunnel excavation and reinforcement was simulated to provide guidance for sensor installation. Then, the FBG strain sensors obtained the stress and strain changes during the reinforcement period and provided a reference for the stability evaluation of the tunnel.

## 2. Tunnel Defects and Comprehensive Prospecting Diagnosis

**Figure 1:**
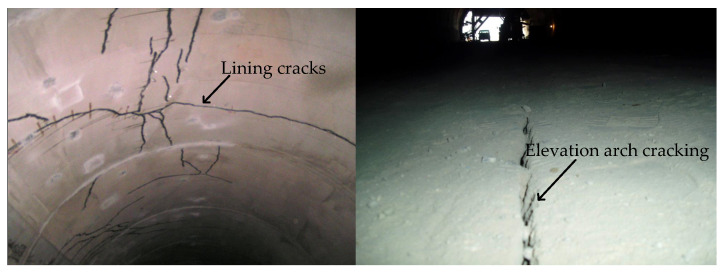
The geological disaster failure of Feiyuze tunnel.

The Feiyuze tunnel is situated in the mountainous region of southeastern Yunnan Province, China. This monolithic, continuous arch tunnel includes a center wall and measures 215 m in length, with 23.4 m net width, and stands at an elevation of 1377 m. The burial depth of the tunnel reaches 37 m, and it has a longitudinal slope gradient of −0.7%. However, during the construction process, the tunnel experienced cracks and other structural issues in the arch crown and elevation arch, which had a detrimental effect on both the quality of the tunnel and the safety of the construction process, as shown in Figure 1.

**Figure 2:**
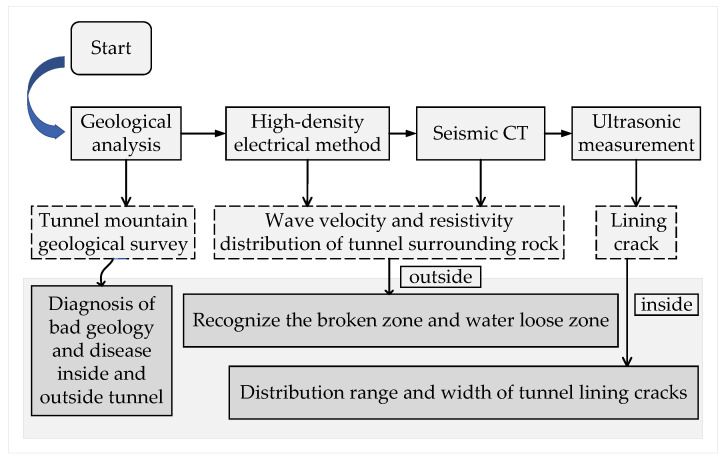
Tunnel geological exploration program.

A comprehensive geophysical exploration plan has been established to accurately identify the causes of tunnel diseases and evaluate the structural stability of the Feiyuze tunnel. Since seismic waves and resistivity are sensitive to physical parameters such as rock strength, water content, and porosity, the characteristics of the tunnel structure are analyzed using seismic CT, the high-density electrical method, and ultrasonic measurement. These methods comprehensively detect tunnel diseases, the geological environment, and fault zones from the external mountainous terrain to the internal lining of the tunnel in Figure 2.

The external environment of the tunnel is investigated using a combination of seismic CT and the high-density electrical method to effectively identify the fragmentation, water content. The high-density electrical method is an array-exploration method that forms an artificial electric field using a Wenner device [19]. Differences in the type, composition, humidity, and temperature of rock and soil masses can be explained by changes in resistivity in the straight or horizontal direction of a vertical profile. Additionally, seismic CT employs seismic waves to penetrate the geological body and create an image of its structure by measuring energy attenuation. Dense rock masses show minimal energy attenuation and high wave velocity, whereas broken rock and soil masses exhibit considerable energy attenuation and low wave velocity [20,21].

**Figure 3:**
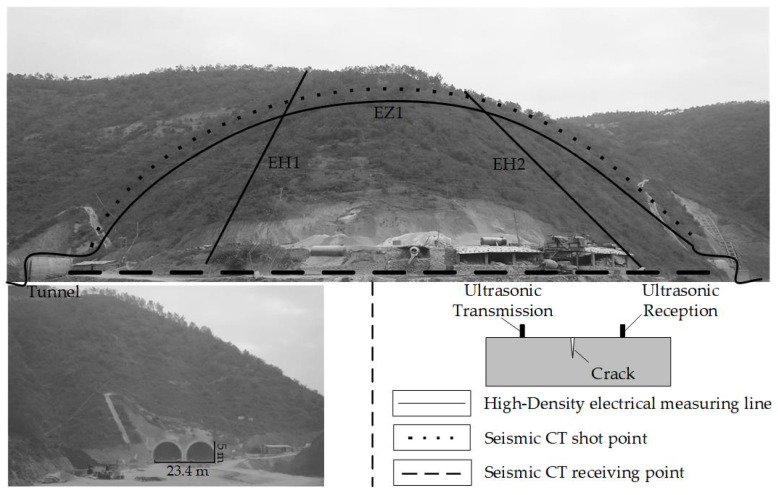
Seismic CT and high-density electrical detection line layout.

In the high-density electrical method employed in this study, there are 3 sections with a total length of 860 m, a total of 328 electrodes, and a detection depth of 90 m. Among these, the EZ1 measuring line is laid along the central axis of the tunnel to detect the apparent resistivity distribution of the mountain in the front view direction, and the EH1 and EH2 measuring lines are laid perpendicular to the central axis of the tunnel to detect the lateral resistivity distribution of the mountain. Then, the geophones are arranged on the vault of the tunnel to receive seismic wave signals, and the exploration measuring line is shown in Figure 3. In the tunnel, the ultrasonic receiver testing point and the transmitter testing point are arranged on the surface of second lining. The width and depth of the concrete crack are obtained by calculating the propagation time of the ultrasonic wave in the concrete [22,23].

For ultrasonic measurement, the measurement points are arranged at both ends of the crack in a spanning and non-spanning manner. The results reveal that there are 24 prominent cracks in the tunnel, with crack widths ranging from 0.6 mm to 1.6 mm. Among these cracks, the majority of them are located in the arch shoulders of the tunnel. In addition, the depth of the cracks in the tunnel varies from 114 mm to 459 mm, which is also a matter of concern and reflects the risk of further destabilization of the structural defects. The distribution of crack width and depth is shown in Table 1.

**Figure 4:**
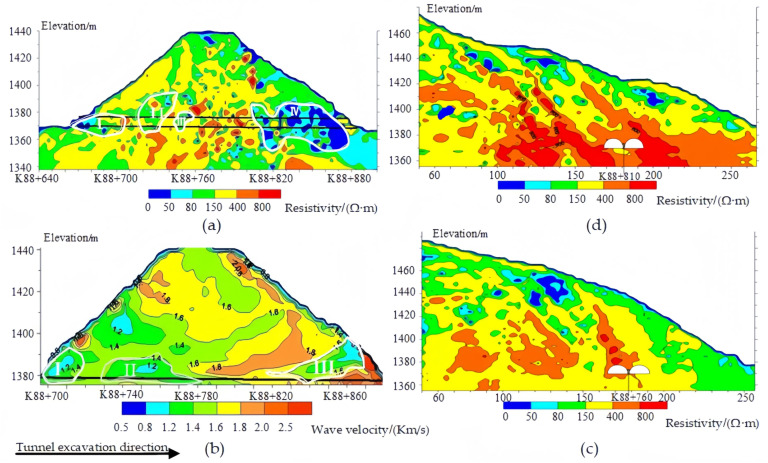
Comprehensive geophysical exploration of Feiyuze tunnel. (**a**) EZ1 profile apparent resistivity distribution image. (**b**) Tunnel seismic CT wave velocity image. (**c**) EH2 profile apparent resistivity distribution image. (**d**) EH1 profile apparent resistivity distribution image.

Seismic CT and high-density electrical resistivity imaging reveal the existence of three low-velocity anomaly zones along the tunnel axis, which correspond to three rock fracture zones, as shown in Figure 4. The first anomaly zone is located between K88+690 and K88+710, with a velocity less than 1.4 Km/s, locally less than 1.2 Km/s, and it corresponds to the first low resistivity anomaly position, with a rock fracture zone width of 20 m. The second low-velocity anomaly zone is situated between K88+715 and K88+770, with a velocity less than 1.4 Km/s, locally less than 1.2 Km/s, and it corresponds to the second and third low resistivity anomaly positions, with a rock fracture zone width of 55 m. The third low-velocity anomaly zone is located between K88+810 and K88+860, with velocity ranging from 1.6 Km/s to 1.2 Km/s, and it corresponds to the fourth low resistivity anomaly position, with a rock fracture zone width of 50 m. These anomalous areas are closely related to the location of the lining crack distribution inside the tunnel.

Through an analysis of the mechanical wave velocity and resistivity of the peripheral rock in the tunnel, the rock is classified as Class IV and characterized by extensive weathering, dense rock fissures, and the presence of fracture zones. Based on the comprehensive geophysical exploration for the diagnosis of tunnel defects, we infer that the main causes of tunnel issues are mountain bias and the redistribution of excavation-induced stress. Thus, the combination of various geophysical exploration methods can provide scientific evidence for tunnel repair and surrounding rock reinforcement.

## 3. Force Analysis of CFRP Strengthening Tunnel Lining

The complex geological conditions in mountainous areas make controlling tunnel fracture cracking and implementing reasonable measures to regulate developed cracks challenging aspects and hotspots in the construction process. Sprayed concrete, crack embedded filling, profiled bar embedded reinforcement, arch surround reinforcement and the removable arch are common methods of reinforcement. However, these methods have the disadvantages of slow construction and intrusion into the building boundaries and other deficiencies.

In view of the crack distribution characteristics of the Feiyuze tunnel, carbon fiber brings new ideas for crack reinforcement. CFRP is a high-performance material with several fundamental properties, including high strength, stiffness, lightweight, and corrosion resistance. On the one hand, carbon fiber wires work together [24] and greatly improve the overall tensile strength of carbon fiber cloth. On the other hand, by attaching the carbon fibers to the surface of the tunnel structure through epoxy resin, the carbon fibers and the concrete structure will work together to resist cracking and counteract the tensile forces, thereby enhancing and improving the structural mechanical properties. To determine the optimal sensor installation position following CFRP strengthening and acquire reliable monitoring data, finite element analysis tools are employed to analyze the stress field of the rock and lining during tunnel excavation. Based on the results of the comprehensive physical exploration, the parameters of the numerical simulation are shown in Table 2.

**Figure 5:**
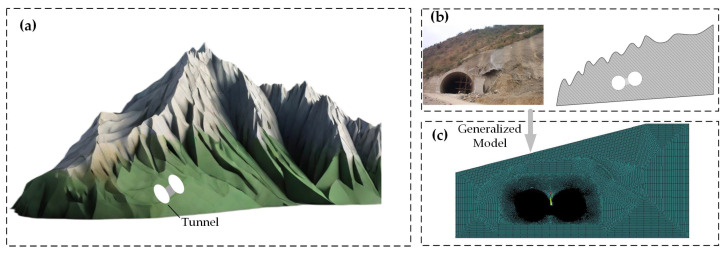
Tunnel excavation modeling program. (**a**) 3D topographic image. (**b**) Topographic profile. (**c**) Model mesh image.

It is assumed that the stress–strain of all materials varies within the elastic-plastic range, and spatial effects are not considered. The surrounding rock and CFRP were simulated by the plane strain Plane 42 and Plane 182 units, respectively, and the lining support was simulated by the Beam 3 unit. These materials exhibit homogeneity, continuity, and isotropy. The Mohr–Coulomb linear elastic constitutive model was selected for both the surrounding rock material and the supporting structure. This model enables effective analysis of the stress–strain behavior and deformation of the soil mass in the underground structure. During tunnel excavation, displacement constraints in the X and Y directions are imposed at the base of the mountain, and the initial stresses are induced by the gravitational force of the soil. The alternate excavation method is employed based on the specific on-site tunnel excavation conditions to effectively control tunnel deformation and ensure the stability of the surrounding rock. The excavation sequence involves initial excavation of the left tunnel, followed by the construction of a central partition wall, and subsequent excavation of the right tunnel. The modeling scheme was built as shown in Figure 5.

**Figure 6:**
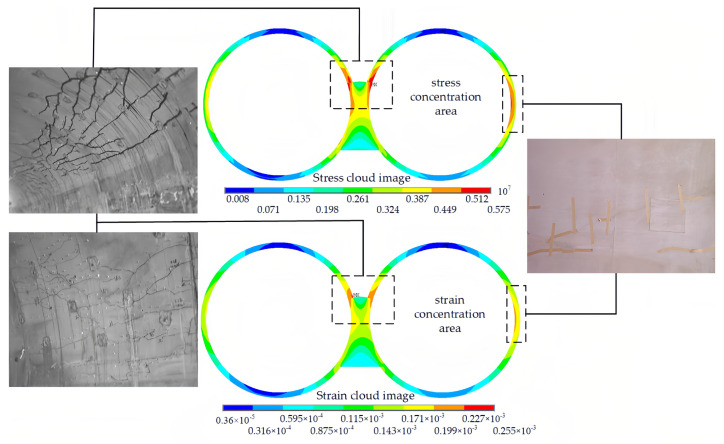
Tunnel excavation stress–strain cloud.

With the stress release during the excavation process, the disturbance range continues to expand, resulting in the redistribution of the stress field in the surrounding rock. In the simulation process, the stress–strain concentration zone appears at the arch shoulder near the middle partition wall, which corresponds to the location where defects occur during actual tunnel excavation. On the one hand, the holes excavated in advance during the construction of the continuous arch tunnel often bear greater stress. This is because the holes excavated in advance are subjected to the soil pressure from subsequent excavations during the excavation process, resulting in greater stress on the tunnel walls. On the other hand, due to the self-weight of the mountain and the surface load, greater pressure is exerted on the tunnel walls closer to the mountainside, as shown in Figure 6.

**Figure 7:**
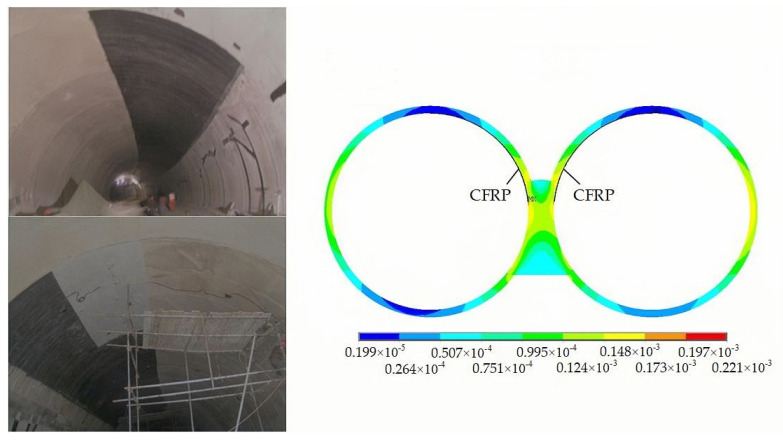
Carbon fiber strengthening lining strain cloud.

By adhering carbon fiber fabric onto the surface of the damaged lining, a composite structure is formed, as shown in Figure 7. The involvement of carbon fiber fabric in bearing the load places certain constraints on the deformation of the concrete lining. Before reinforcement, the average tensile strain of the tunnel arch shoulder is 199 με, while, after reinforcement, it is 148 με, a reduction of nearly 25.6%.

From the overall model calculation, the maximum point of strain before and after reinforcement still appears near the stress concentration area, and the strain suffered by the lining decreases from 255 με to 221 με, which is reduced by 13%. Thus, CFRP fabric as a tensile material can effectively control the expansion of cracks.

**Figure 8:**
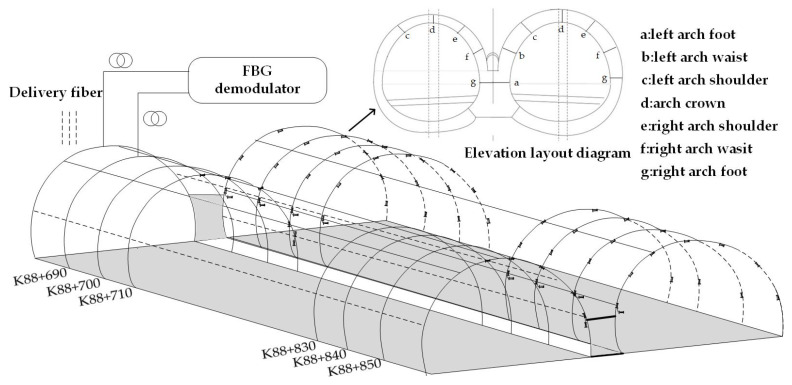
Tunnel section sensor distribution image.

In the actual treatment of tunnel crack reinforcement, it is necessary to first polish the surface of the concrete crack and remove surface floating slurry, oil, and other impurities. On a clean concrete surface, carbon fiber cloth is applied to the damaged area of the lining using epoxy resin. At the same time, it is ensured that the carbon fiber cloth is in full contact with the surface to eliminate any excess bubbles. After curing over a period of time, carbon fiber and concrete form a structural complex. In Figure 8, the mounting position of the sensor is determined based on the analysis of the tunnel strain cloud image. These FBG sensors are set on twenty monitoring sections of the right tunnel every 10 m, where seven FBG strain sensors are separately installed on the arch crown, arch shoulder, and side walls on each cross-section. In addition, the tunnel on the left has four monitoring sections, each section has five monitoring points, and a total of twenty FBG strain sensors are installed. The temperature sensor is connected to the last strain sensor of each section to achieve temperature compensation.

## 4. Analysis of Monitoring Results of Tunnel Lining Strain

**Figure 9:**
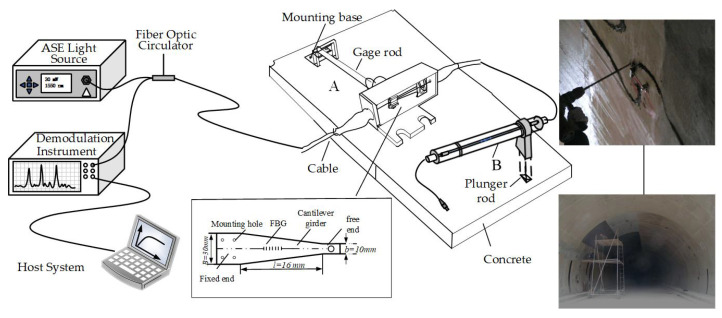
Fiber grating strain detection system and on-site installation.

The strain measurement system comprises an amplified spontaneous emission (ASE) broadband light source, a strain sensor, a fiber grating demodulator, and a host system. The output power of the ASE broadband light source is 10 mW, the spectral flatness is less than 2 db, and the wavelength range is 1515∼1595 nm. The wavelength demodulation accuracy of the optical signal demodulation equipment is 1 pm, the sampling frequency is 2 kHz, the wavelength repetition rate can reach less than 3 pm, and the wavelength demodulation range matches the ASE broadband light source. This light is injected into two FBG sensors, namely FBG sensor A and FBG sensor B, through a circulator. The purpose of this setup is to measure structural strain and provide temperature compensation. The strain monitoring system and on-site installation are shown in Figure 9.

**Figure 10:**
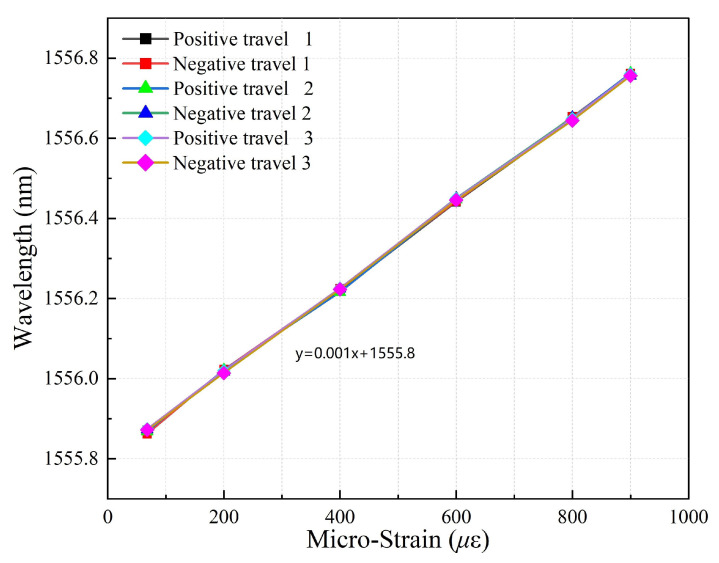
Strain calibration plot of experiment.

The deformation of the liner compresses the deflection and bending of the free end of the cantilever beam, resulting a change in deflection in the measuring rod of the sensor. This change will drive the wavelength shift of the Bragg grating fixed to surfaces of the cantilever beam. The strain of the cantilever $\varepsilon^{\prime }$ can be expressed as:

(1) $$\left\{\begin{array}{c} \omega =\frac{4Fl^{3}}{Ebh^{2}}\\ \varepsilon^{\prime }=\frac{h}{l}\omega \end{array}\right.$$

where *l* and *h* are the length and thickness of the equal-strength cantilever beam, respectively. *L* is the length of the gauge rod. *E* is the elastic modulus. *F* is the force on the cantilever. According to Equation (1), the detected applied strain on the second lining can be expressed as:

(2) $$\left\{\begin{array}{c} \frac{\Delta \lambda_{B}}{\lambda_{B}}=\left(1-p_{e}\right)\frac{4kl^{2}}{Ebh^{2}}\Delta l\\ \varepsilon =\frac{\Delta l}{L} \end{array}\right.$$

where $\Delta \lambda_{B}/\lambda_{B}$ is the relative wavelengths, $p_{e}$ is the effective strain-optic constant for the fiber, *k* is the elastic coefficient of the measuring rod, and Δl is the elongation. FBG sensor A is used to measure the structure axial strain, and FBG sensor B is used to measure the temperature. $\Delta \lambda_{B}$ is the Bragg wavelength shift of sensor A, $\Delta \lambda_{T}$ is the Bragg wavelength shift of sensor B, and $\Delta \lambda_{T}$ can be described as:

(3) $$\left\{\begin{array}{c} \frac{\Delta \lambda_{T}}{\Delta T}=S_{T}C_{g,T}+S_{\epsilon }C_{g,\epsilon }C_{g,T}\left(\alpha_{Fe}-\alpha \right)\\ \varepsilon_{T}=\frac{\Delta \lambda_{T}}{S_{T}^{T}}\alpha_{H} \end{array}\right.$$

where ΔT represents the temperature variation. $\alpha_{Fe}$ is the thermal expansion coefficient of the steel pipe, and usually its value is 11.8 × 10^−6^ °C^−1^. α = 0.55 × 10^−6^ °C^−1^ is the thermal expansion coefficient of the fiber, and $\alpha_{H}$ is the thermal expansion coefficient of the structure and is determined by the physical parameters of the structure. $C_{g,\epsilon }$≈ 0.85 is the strain transmission coefficient between the fiber and host structure, and $C_{g,T}$≈ 0.94 is the thermal transmission coefficient of the encapsulated FBG. $S_{T}^{T}$ = $S_{T}C_{g,T}+S_{\epsilon }C_{g,\epsilon }C_{g,T}\left(\alpha_{Fe}-\alpha \right)$ ≈ 20.56 pm°C^−1^ is the thermal sensitivities of the FBG sensor B suspended on the surface of the structure. In the strain calibration experiment, a linear relationship is derived based on the principle of least squares, as shown in Figure 10.

**Figure 11:**
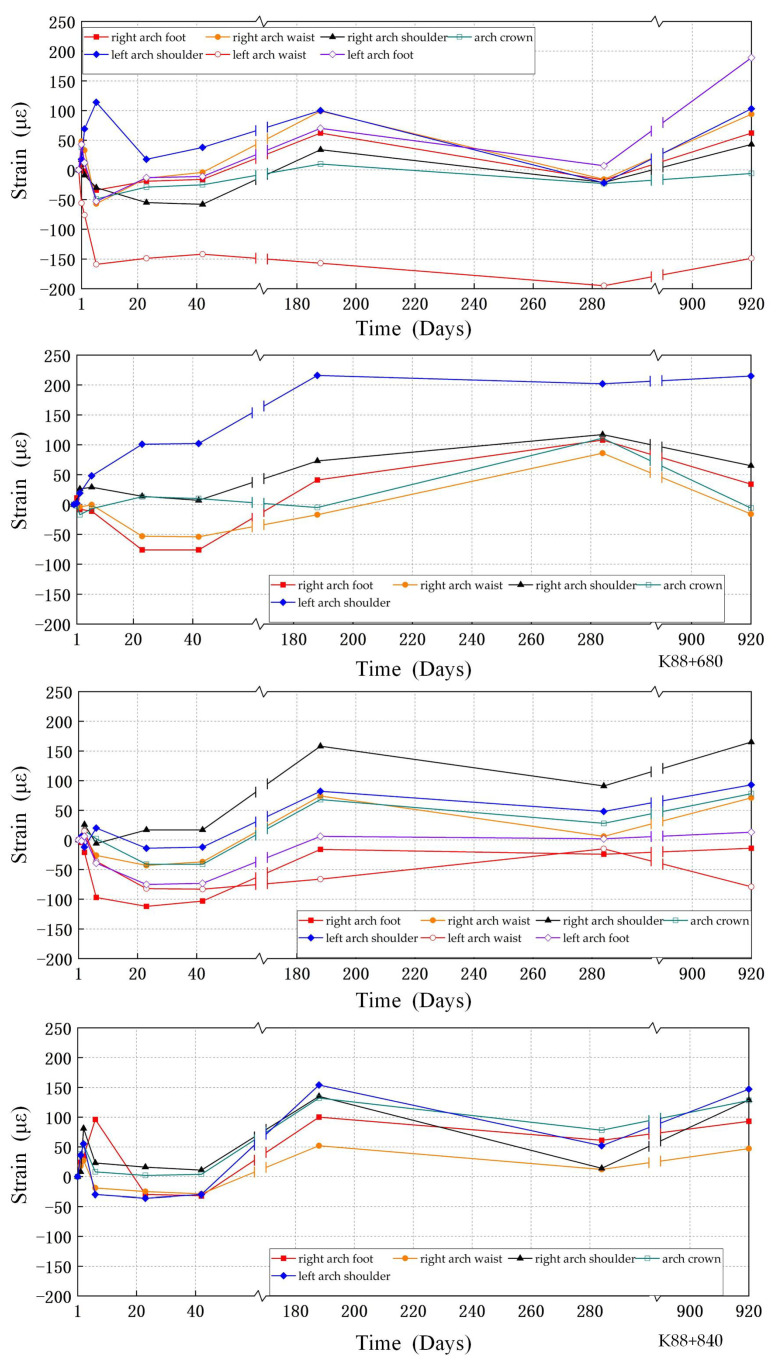
Strain at cross-sections of K88+680 and K88+840 sections.

In the strain calibration experiments, the change in wavelength of the sensor is recorded by applying different loads at both ends of the sensor and performing multiple experiments with positive and negative stress. In the strain variation, the micro-strain of the sensor is the x-axis, and the center wavelength of the fiber grating is the y-axis. The linear relationship is derived from the principle of least squares. According to the experimental data, the strain sensitivity coefficient of the FBG strain sensor is 1.060 pm/με, the nonlinear error is 0.2%, and the repeatability error is 0.52%. The strain values of the 24 tunnel sections are obtained through monitoring using FBG strain sensors. During a period of 920 days, four monitoring sessions were conducted during the construction phase, and another four were conducted during the operational phase. The test results of two typical cross-sections K88+680 and K88+840 were taken for analysis, as shown in Figure 11.

For lining cracking, more attention is paid to the cracks caused by the tension of the structure. The long monitoring data show that the strain amplitudes of the two tunnel lining sections are between −200 με and 250 με. The tensile strain values of the two sections show an overall decreasing trend, but the deformation characteristics of some areas still exhibit tensile strain. The maximum tensile strains for both tunnel sections occur at the arch waist of the right tunnel and are −195 με and −79 με, respectively. According to the simulation results, this location is located in the stress–strain concentration area. This phenomenon is due to the fact that the arch shoulder and arch waist of a continuous arch tunnel are subjected to loads from the side wall and the middle partition wall, resulting in a higher concentration of stress in these two areas. Furthermore, in the left tunnel of the continuous arch tunnel, the point of maximum strain occurs at the shoulder of the arch is 215 με and 147 με, respectively. This is because the mountain pressure will be redistributed when the adjacent cave is excavated. At this time, the arch crown of the left cave will be supported by the upper load, while the foot of the arch will be pressurized upwards, and therefore the arch shoulder will be subjected to compressive strain. Therefore, the arch shoulder and arch waist of the tunnel are more important areas for monitoring.

**Figure 12:**
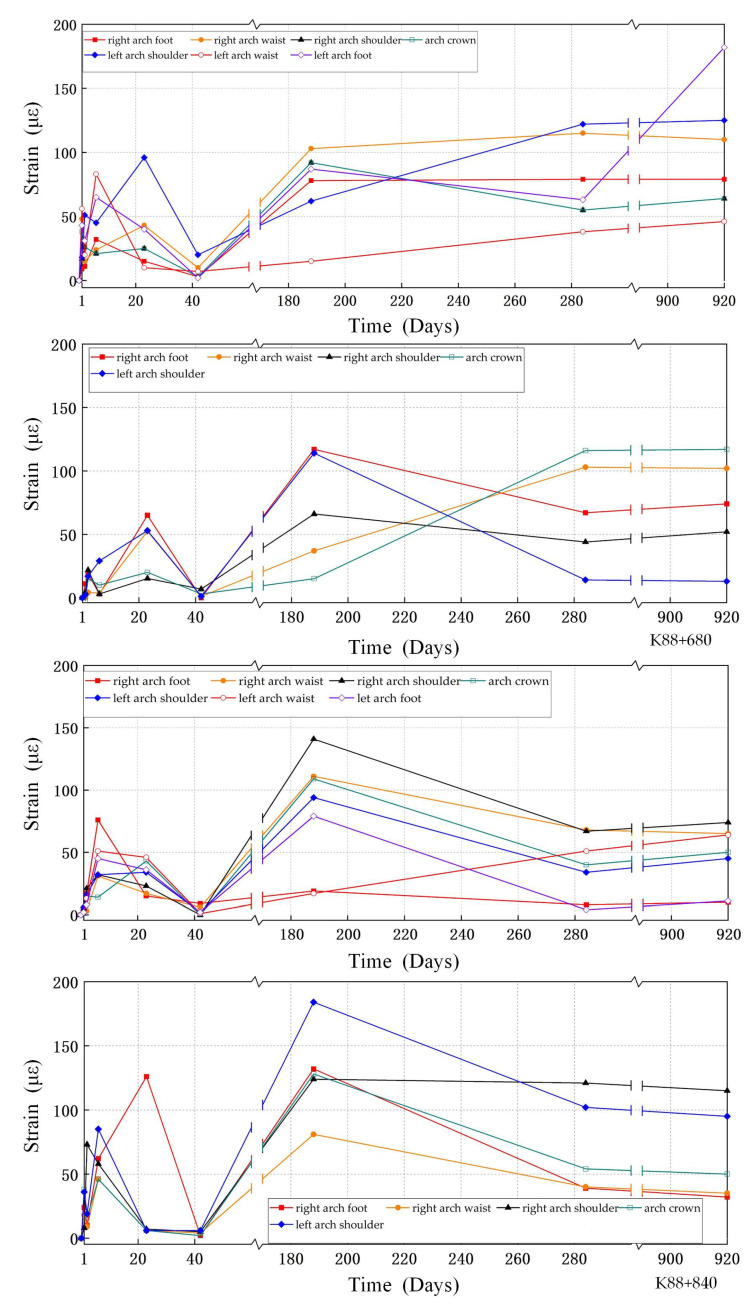
Strain difference of K88+680 and K88+840 sections.

The measurement results of two typical sections, K88+680 and K88+840, were taken for analysis, as shown in Figure 12. During the measurement, the trend of strain difference at each monitoring point of the two sections is consistent. The maximum strain difference of the two cross-sections are 182 με and 184 με, respectively. Due to the differences in material properties and strain capacity between the lining structure and the CFRP, there will be a phase of stress adjustment. Over time, the material will gradually adapt to these stresses and form a composite with the structure. At this stage, both sections show unstable fluctuations, with the maximum strain difference of 72 με in section K88+680 and 76 με in section K88+840. It is worth noting that on day 42, there were significant fluctuations at all monitoring points in the tunnel. This is due to the fact that the lining structure will be subjected to additional vehicle loads once the tunnel is in operation. These pressures will result in strains on the lining surface, which will increase the strain values. In the subsequent monitoring, the strain fluctuations remained within a stable range. No new cracking or crack extension phenomena have occurred in the tunnel lining, and there is no evidence of peel or fracture damage on the surface of the carbon fiber. This indicates that CFRP has effectively controlled structural deformation. The external force will cause the elastic deformation of the structure, and the CFRP and concrete pasted on the lining surface continuously adjust the structural stress and restrain its deformation behavior.

The above studies have shown that FBG strain sensors installed on the surface of the tunnel lining can monitor the strain behavior of the tunnel lining in real time. By analyzing the monitoring data, the safety of the tunnel structure can be assessed. The monitoring results of 920 days show that the change in the strain of the second lining tends to be stable. Therefore, the combined detection of physical exploration and FBG sensors for the structure provided an effective monitoring method for evaluating tunnel stability.

## 5. Discussion and Conclusions

In underground engineering, excavation or blasting can cause deformation, fracture, or even instability in the surrounding rock. Thus, the monitoring of the lining is of utmost importance for ensuring the safety of the tunnel. This study links geophysical exploration, simulation, and sensor monitoring and provides a reliable method for tunnel engineering safety monitoring. Firstly, a comprehensive interpretation method based on geological defects is proposed. By interpreting the physical parameters, a tunnel simulation model has been established. Finally, strain of the second lining of the Feiyuze tunnel is monitored using FBG strain sensors. The main conclusions of this study can be summarized as follows.

(1) Combined with the geological environment of the continuous arch tunnel, a corresponding comprehensive physical exploration program is proposed to analyze the mountain structure and tunnel lining diseases.

(2) The wave velocity of the tunnel surrounding rock is in the range of 1.4 to 1.8 Km/s, and the distribution of rock integrity is uneven, belonging to the class IV rock. The main causes of tunnel issues are mountain bias and the redistribution of excavation-induced stress, and the lining cracks all appeared at the arch shoulder of the tunnel. Based on this, the stress–strain field of continuous arch tunnel excavation and reinforcement was simulated numerically.

(3) The simulation results show that the stress concentration area of the tunnel corresponds to the actual location of cracking disaster, which verifies the reliability and validity of the simulation model and provides a theoretical basis for the sensor monitoring program. The strain cloud image shows that the average tensile strain of the tunnel arch shoulder is 199 με, while after reinforcement it is 148 με, a reduction of nearly 25.6%. Thus, CFRP plays an essential role in controlling lining deformation.

(4) During the monitoring period, the strain amplitudes of the two tunnel lining sections are between −200 με and 250 με. In the preliminary monitoring, the lining structure and CFRP adjust stress distribution under external force, and the strain value increases, then decreases, and eventually stabilizes. The maximum strain values of K88+680 and K88+840 sections are −153 με and −112 με, respectively. In addition, the lining cracks do not show any expansion behavior. Therefore, the combination of comprehensive physical exploration and FBG sensors provides an effective monitoring method for tunnel stability evaluation.

## Figures and Tables

**Table 1:** The size characteristics of second lining cracks in the tunnel.

Position	Width (mm)	Depth (mm)	Position	Width (mm)	Depth (mm)
K88+682	1.00	297	K88+735	0.84	252
K88+696	0.60	188	K88+742	1.40	459
K88+705	0.60	289	K88+751	1.00	450
K88+708	1.20 0.70	331 365	K88+754	1.00	335
K88+714	0.80 1.00	163 127	K88+764	1.00	401
K88+718	1.04	326	K88+775	1.00	354
K88+723	1.44	423	K88+781	0.88	290
K88+728	1.60 1.60 1.20	360 437 114	K88+817	1.00	328
K88+824	0.80	214	K88+811	1.00	311
K88+833	1.00	195	K88+833	1.00	232

**Table 2:** Calculation parameters of the materials.

Material Name	Elastic Modulus	Density	Poisson’s Ratio	Fiction Angle
	**(E/GPa)**	**(Kg/m^3^)**		**(Θ/°)**
IV Grade surrounding rock	4.95	2.20 × 10^3^	0.32	37
Tunnel lining	30	2.36 × 10^3^	0.20	54
CFRP	235	1.75 × 10^3^	0.26	-

## Data Availability

Data are contained within the article.
